# α-Synuclein Regulates Peripheral Insulin Secretion and Glucose Transport

**DOI:** 10.3389/fnagi.2021.665348

**Published:** 2021-07-30

**Authors:** Nadeeja Wijesekara, Rosemary Ahrens, Ling Wu, Tammy Langman, Anurag Tandon, Paul E. Fraser

**Affiliations:** ^1^Tanz Centre for Research in Neurodegenerative Diseases, University of Toronto, Toronto, ON, Canada; ^2^Department of Medicine, University of Toronto, Toronto, ON, Canada; ^3^Department of Medical Biophysics, University of Toronto, Toronto, ON, Canada

**Keywords:** α-synuclein, GLUT4, diabetes, insulin secretion, Parkinson’s disease

## Abstract

**Aim:**

Population based studies indicate a positive association between type 2 diabetes (T2D) and Parkinson’s disease (PD) where there is an increased risk of developing PD in patients with T2D. PD is characterized by the abnormal accumulation of intraneuronal aggregated α-synuclein (α-syn) in Lewy bodies, which negatively impact neuronal viability. α-syn is also expressed in both pancreatic islets and skeletal muscle, key players in glucose regulation. Therefore, we examined the functional role of α-syn in these tissues.

**Methods:**

Using mice lacking, overexpressing or transiently injected with α-syn, effects on glucose and insulin tolerance and insulin secretion were determined, with further characterization of the effects on GLUT4 translocation using GLUT4*myc* myotubes.

**Results:**

Mice genetically ablated for α-syn became glucose intolerant and insulin resistant with hyperinsulinemia and reduced glucose-stimulated insulin secretion (GSIS). Mice overexpressing human α-syn are more insulin senstive and glucose tolerant compared to controls with increased GSIS. Injection of purified α-syn monomers also led to improved glucose tolerance and insulin sensitivity with hightened GSIS. α-syn monomer treatments increased surface GLUT4 levels in myotubes but without any significant change in Akt phosphorylation. The increase in cell surface GLUT4 was largely due to a large reduction in GLUT4 endocytosis, however, with a compensatory reduction in GLUT4 exocytosis.

**Conclusion:**

Cumulatively, this data suggests that α-syn modulates both pancreatic beta cell function and glucose transport in peripheral tissues, thereby playing a pivitol role in the maintenance of normal glucose homeostasis.

## Introduction

Type 2 diabetes (T2D) is associated with Parkinson’s Disease (PD) and can be impacted by other comorbidity diseases such as diabetes (Hu et al., 2007; D’Amelio et al., 2009; Schernhammer et al., 2011; Xu et al., 2011; De Pablo-Fernandez et al., 2017). It has been reported that 50–80% of PD patients have abnormal glucose tolerance (Sandyk, 1993). Compared to non-diabetic individuals, patients with T2D have increased risk up to 38% and a hazard ratio of 1.32 for developing PD (Yue et al., 2016; De Pablo-Fernandez et al., 2018) and it has been shown that patients with diabetes who develop PD have a more aggressive form of the disease (Foltynie et al., 2018). Studies have indicated that nearly 60% of non-diabetic people with PD are insulin resistant (Hogg et al., 2018). In addition, common anti-diabetic treatments such as dipeptidyl peptidase-4 (DPP4) inhibitors, glucagon-like peptide-1 (GLP-1) agonists and metformin have shown promise in the treatment of PD and cognitive impairment in human and animal models of PD (Ashraghi et al., 2016).

PD is the most common movement neurodegenerative disorder with progressive degeneration of dopaminergic neurons in the substantia nigra. The pathophysiological mechanisms underlying PD are still largely unknown, however, it is characterized by the abnormal accumulation of intraneuronal α-synuclein (α-syn) aggregates in Lewy bodies, which negatively impact neuronal viability (Ozansoy and Başak, 2013). Missense mutations in α-syn are associated with familial PD and posttranslational modifications, particularly phosphorylation of Serine-129 (pS129), are considered critical to progression of the disease (Polymeropoulos, 2000; Stewart et al., 2015). These changes impact normal α-syn functions related to neurotransmitter synthesis and regulation of exocytosis (Perez et al., 2002; Larsen et al., 2006).

Recently, pathological pS129-positive α-syn deposits were found in the pancreatic beta cells of 93% of subjects with PD, in 68% of subjects with T2D following neuropathological examination and in 17% of control subjects (Martinez-Valbuena et al., 2018). Further, increased accumulation, aggregation, and phosphorylation of α-syn was observed in the pancreatic islets of non-human primate models of spontaneous T2D (Sun et al., 2020). Serum α-syn levels were also inversely correlated with insulin resistance indicators such as body mass index, homeostatic model assessment for IR (HOMA-IR) and immunoreactive insulin (Rodriguez-Araujo et al., 2015). These studies together suggest that α-syn may be important for pancreatic beta cell function and regulation of insulin sensitivity. These findings support links between α-syn and T2D which is characterized by both a loss of insulin sensitivty in peripheral tissues such as skeletal muscle and impaired insulin secretion by pancreatic beta cells.

In the current study, glucose metabolism was examined in mice lacking or, overexpressing or injected with α-syn in order to understand its physiological role in metabolic pathways. Our data indicates α-syn regulates both glucose-stimulated insulin secretion (GSIS) in beta cells and glucose transport in skeletal muscle. The observed effects on glucose tolerance and insulin sensitivity, point to a significant role for α-syn in the regulation of glucose homeostasis.

## Materials and Methods

### Animal Care

All experiments were approved by the Animal Care Committee at the University Health Network. α-syn knockout mice B6;129 × 1-*Snca^TM 1*Rosl*^*/J were obtained from Jackson Labs (Bar Harbor, ME, United States) (Abeliovich et al., 2000). Both the knockout and control animals are of 50% C57 and 50% 129 background and were age-matched. Mice were characterized at 3 months of age. Human wild type α-syn overexpressing mice of FVB background strain were generated using a hamster prion protein promoter and littermate controls were used (Chen et al., 2013). Mice were characterized at 4 months of age. 3-Month old C57BL6J mice were used for α-syn treatments and for examining α-syn expression. Male mice fed a chow diet were used for all experiments.

### α-Syn Monomer Treatment

Human α-syn monomers were expressed and extracted from bacteria as previously described (Jo et al., 2000). 50 μg of α-syn monomer was injected intraperitoneally in a 100 μL volume at 6 weeks of age, followed by a second injection after 2 weeks. Animals were characterized 4 weeks after second injection at 12 weeks of age.

### Glucose and Insulin Tolerance Tests

*GTT:* Following a 4 h fast, glucose (1.5 g/kg body weight) was given by oral gavage and glucose from tail vein blood was measured at 0, 15, 30, 60, 90, or 120 min using a glucometer. Blood was collected in EDTA-coated microvettes (Sarstedt) at 0 and 15 min and plasma isolated for insulin measurement using an ultrasensitive insulin ELISA (ALPCO Diagnostics). *ITT:* Following 4 h fast, insulin (0.75 IU/kg body weight) was injected intraperitoneally and plasma glucose was measured at 0, 15, 30, and 60 from tail vein blood using a glucometer.

### Islet Isolation and Insulin Secretion

Mouse islets were isolated by collagenase digestion of the pancreas as described (Wijesekara et al., 2010). Insulin secretion was assessed as reported from isolated islets stimulated for 1 h with 2 mmol/L (basal) or 11 mmol/L glucose (Wijesekara et al., 2010). Secreted insulin was quantified by ELISA and normalized to total DNA. Wild type islets were treated *in vitro* in culture medium for 24 h with vehicle (20 mM TrisHCl pH 7.4) or 1.68 μg/mL α-syn monomers prior to and during the insulin assay.

### Cell Culture

L6-GLUT4*myc* myoblasts were seeded onto 12-well (for protein extraction) or 24-well (for GLUT4 translocation) plates and were differentiated into myotubes in α-MEM supplemented with 2% (vol/vol) FBS and 1% (vol/vol) antibiotics/antimycotics. Cells were allowed to fully differentiate into myotubes and were deprived of serum for 3 h prior to any experimental manipulation. For cell lysate preparation, myotubes were stimulated for 10 min in α-MEM with vehicle (20 mM TrisHCl pH 7.4), 100 nM insulin [Humulin R] (Eli Lilly Canada) or 1.68 μg/mL α-syn monomers. The cells were lysed with 2X Laemmli sample buffer supplemented with 1 mM DTT and protease inhibitors.

### Western Blot Analysis

Tissue or islet lysates (35 μg total protein) were resolved by 4–10% Tris-Glycine gels and immunoblotted with primary antibody against α-syn [syn-1] (BD Biosciences) [1:1000] and GAPDH (Sigma) [1:1000]. Lysates from myotubes were immunoblotted with primary antibody against phospho-Akt T308 (Cell Signaling) [1:1000] and β-actin (sigma) [1:5000]. Immunoblots were scanned within the linear range and quantified using the computer software NIH Image J. Phospho-Akt is normalized to β-actin and expressed as fold change over vehicle treatment.

### Immunostaining and Microscopy

Isolated pancreata were fixed in formalin, paraffin-embedded and 5 μm sections were cut at 100 μm intervals. Deparaffinized sections were subjected to antigen retrieval in TE buffer and co-stained with syn-1 (BD Biosciences) [1:250] or insulin (Dako) [1:500] primary antibodies. Images were acquired using a Zeiss confocal microscope using Zen software (Carl Zeiss Canada).

### GLUT4 Translocation

The amount of cell surface GLUT4*myc* was determined by an antibody-coupled colorimetric absorbance assay, as was previously described (Wijesekara et al., 2006). Briefly, cells were stimulated for 20 min in a-MEM with vehicle or 1.68 μg/mL α-syn monomers in the presence or absence of 100 nM insulin. Cells were then exposed to polyclonal anti-*myc* antibody (1:100) for 60 min, fixed with 4% paraformaldehyde (PFA) for 10 min, and incubated with peroxidase-conjugated goat anti-rabbit IgG (1:1,000) for 1 h. Cells were washed six times and 1 ml of OPD reagent (0.4 mg/ml o-phenylenediamine dihydrochloride and 0.4 mg/ml urea hydrogen peroxide) was added for 30 min at room temperature. The reaction was stopped with 0.25 ml of 3 N HCl. Optical absorbance of the supernatant was measured at 492 nm. Background, as measured in samples incubated in peroxidase-conjugated anti-rabbit IgG alone (without primary antibody), was subtracted from all values.

### Measurement of GLUT4*myc* Recycling

The rate of GLUT4myc recycling was determined as described (Wijesekara et al., 2006). Briefly, at 37°C, cells were incubated in the absence or presence of α-syn or insulin while being exposed to polyclonal anti-*myc* antibody for the times indicated. At each time point, cells were fixed with 4% paraformaldehyde for 10 min, permeabilized with 0.1% TritonX-100 for 15 min, and reacted with peroxidase-conjugated goat anti-rabbit IgG (1:1000) for 1 h at 4°C to determine the amount of anti-*myc* antibody bound at the cell surface as well as that which had become labeled at the surface and subsequently internalized during the incubation time. 1 mL of OPD reagent was added for 30 min at room temperature. The reaction was stopped with 0.25 mL of 3 M HCl. Absorbance of the supernatant was measured at 492 nm. Total amount of GLUT4*myc* was determined by incubating the cells with the anti-*myc* antibody following permeabilization. The amount of GLUT4*myc* labeled at each time point was expressed as a percent of total cellular GLUT4*myc* content.

### Measurement of GLUT4*myc* Internalization

GLUT4*myc* internalization was measured as was previously described (Wijesekara et al., 2006). Briefly, cells were stimulated with or without α-syn or insulin at 37°C for 20 min. Cells were rinsed 3 times with ice-cold PBS and reacted with the polyclonal anti-myc antibody (1:200) at 4°C for 1 h. After washing 2 times with ice-cold PBS, the surface labeled GLUT4*myc* was allowed to internalize by re-warming the cells to 37°C in the presence or absence of α-syn or insulin. At the indicated times, cell plates were placed on ice, washed with ice-cold PBS, fixed with 4% paraformaldehyde for 10 min and incubated with peroxidase-conjugated goat anti-rabbit IgG (1:1000) for 1 h at 4°C. 1 mL of OPD reagent was added for 30 min at room temperature. The reaction was stopped with 0.25 mL of 3 M HCl. Absorbance of the supernatant was measured at 492 nm. The amount of GLUT4*myc* remaining on the cell surface at any time point after re-warming was expressed as a percentage of the cell surface GLUT4*myc* level at 0 min of endocytosis.

### Statistical Analysis

Data are expressed as mean ± SEM. Significance was determined using Student’s *t*-test or one-way or two-way ANOVA with Tukey or Bonfferoni *post hoc* test. *p* < 0.05 was considered statistically significant.

## Results

### α-Syn Is Expressed in Pancreatic Islets and Skeletal Muscle

**FIGURE 1:**
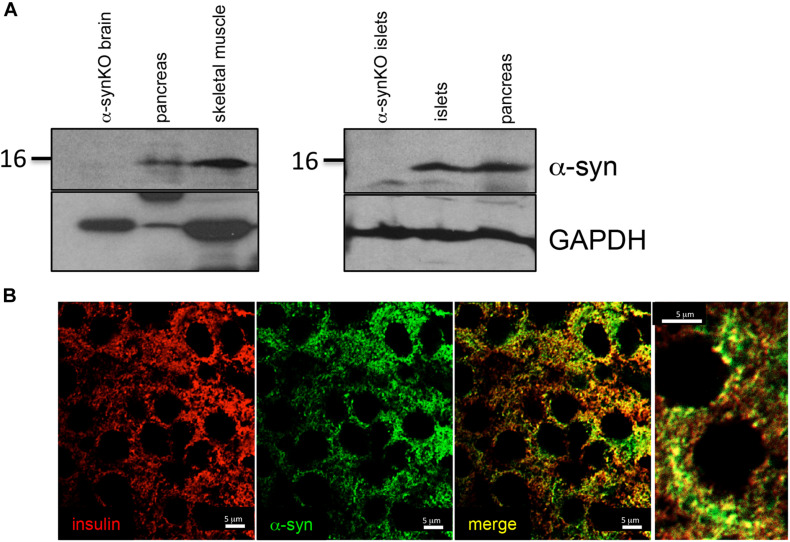
α-syn expressed in tissues regulating glucose homeostasis. **(A)** Western blot analysis of α-syn and GAPDH in mouse pancreas (*n* = 1), skeletal muscle (*n* = 1) and pancreatic islets (*n* = 2) and **(B)** immunostaining for insulin (*red*) and α-syn (*green*) in mouse pancreatic islets.

We observed α-syn expression by immunoblot analysis in total lysates of pancreas, skeletal muscle and isolated pancreatic islets from wild type mice (Figure 1A). Brain and islet lysates from α-syn global knockout (α-synKO) mice were used as negative controls. α-syn was observed to partially co-localize with insulin, confirming its expression in pancreatic beta cells (Figure 1B).

### α-Syn Knockout Mice Are Glucose Intolerant and Insulin Resistant Compared to Controls

**FIGURE 2:**
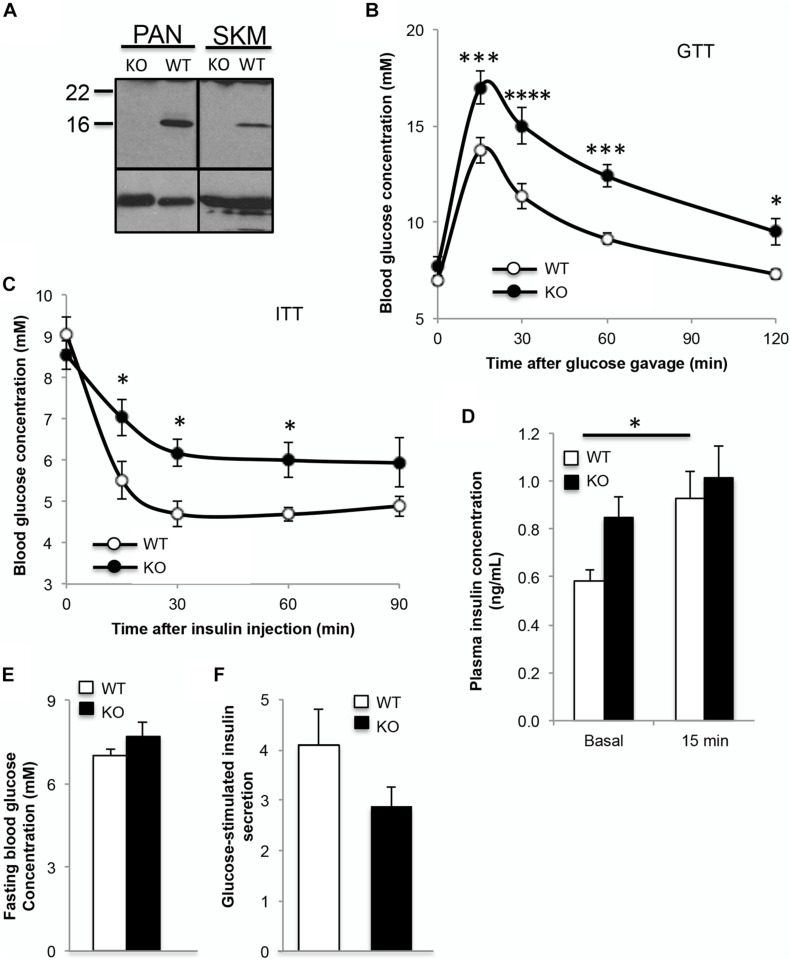
Absence of α-syn leads to glucose intolerance and insulin resistance in mice. **(A)** Western blot analysis showing absence of α-syn in pancreas and skeletal muscle (skm), **(B)** oral glucose tolerance test (*GTT, 1.5g glucose/kg body weight*), **(C)** insulin tolerance test (*ITT, 0.75IU insulin/kg body weight*), **(D)** plasma insulin during GTT (0 and 15 min after glucose gavage), **(E)** fasting blood glucose and **(F)** glucose-stimulated insulin secretion expressed as fold over basal from isolated islets in 3 month old male wild type (WT, *white bars*) and α-syn knockout (KO, *black bars*) mice on chow diet [**(B–E)***: n* = 8–9, **(F)**: *n* = 4, *p < 0.05, ***p < 0.001, ****p < 0.0001].

In order to understand the role of α-syn in glucose metabolism, glucose and insulin tolerance tests were performed in α-synKO mice (Figure 2A). At 3 months of age, α-synKO mice were both glucose intolerant and insulin resistant compared to controls (Figures 2B,C). Fasting plasma insulin was elevated, likely as a response to the insulin resistance, but showed impaired GSIS response, suggesting the presence of beta cell dysfunction in the absence of α-syn (Figure 2D). α-synKO mice maintained body weights (26.1 ± 1.2 vs. 27.3 ± 1.7 g) similar to wild type mice and displayed comparable fasting blood glucose (Figure 2E). This suggests that α-syn is required for proper mainenance of glucose tolerance and insulin senstivity. To confirm that the observed effects are directly linked to α-syn in beta cells, GSIS was measured in isolated islets from α-synKO and wild type mice. *In vitro* glucose-stimulation of α-synKO islets showed a 25% reduction in insulin secretion compared to wild type controls (Figure 2F). These data are consistent with a functional role of α-syn in insulin secretion by pancreatic beta cells.

### Transgenic α-Syn Overexpression and Peripheral Injection of α-Syn Improves Glucose Homeostasis

**FIGURE 3:**
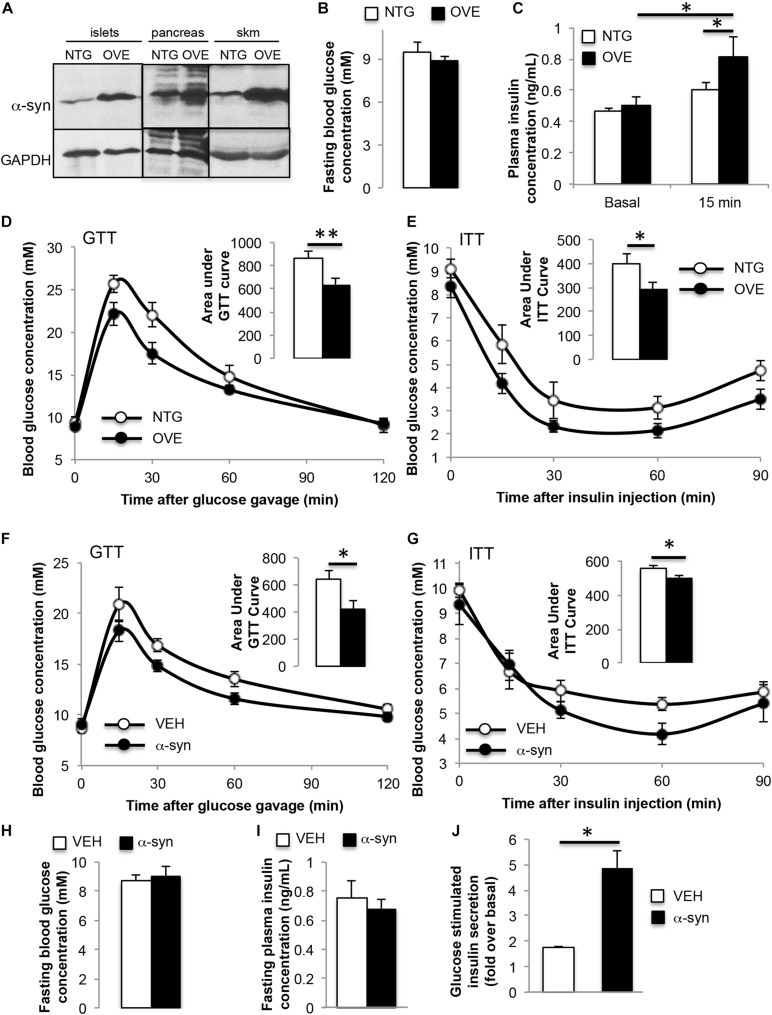
α-syn overexpression or α-syn monomer injection leads to improved glucose tolerance and insulin sensitivity. **(A)** Western blot analysis showing increased expression of α-syn in transgenic islets, pancreas and skeletal muscle (skm), **(B)** fasting blood glucose, **(C)** plasma insulin during oral glucose tolerance test (GTT, 1.5 g glucose/kg body weight) at 0 and 15 min after glucose gavage, **(D)** GTT and **(E)** insulin tolerance test (ITT, 0.75 IU insulin/kg body weight) in 4-month old male non-transgenic (NTG, *white bars*) and α-syn overexpressing (OVE, *black bars*) mice on chow diet [**(B,D,E)**: *n* = 6, **(C)**: *n* = 7–10, ^∗^*p* < 0.05, ^∗∗^*p* < 0.01]. **(F)** Oral glucose tolerance test (GTT, 1.5 g glucose/kg body weight), **(G)** insulin tolerance test (ITT, 0.75 IU insulin/kg body weight), **(H)** fasting blood glucose, and **(I)** fasting plasma insulin 4 weeks after vehicle (VEH, *white bars*) or α-syn monomer (α-syn, *black bars*) injections in male wild type mice on chow diet [**(F–I)**: *n* = 7, ^∗^*p* < 0.05]. Mice were treated with two intraperitoneal injections of 50 μg of α-syn at 2 weeks apart. **(J)** Glucose-stimulated insulin secretion expressed as fold over basal from wild type islets treated with vehicle, or 1.68 μg/mL α-syn monomers for 24 h (*n* = 4, ^∗^*p* < 0.05).

In contrast to α-syn knockout, trangenic mice overexpressing human α-syn at 4 months of age showed improved glucose tolerance and insulin sensitivity, with body weight (24.5 ± 0.5 vs. 24.3 ± 0.5 g), fasting blood glucose and fasting plasma insulin similar to non-transgenic controls (Figure 3A–E). GSIS was significantly elevated in the α-syn overexpressing mice (Figure 3C). In order to further understand the impact of α-syn, wild type mice were subjected to two intraperitoneal injections of recombinant, purified α-syn monomers at 2 week intervals, starting at 6 weeks of age. Four weeks after the second injection (i.e., 12-weeks of age), α-syn injected animals exhibited improvements in glucose tolerance and insulin sensitivity compared to vehicle only injected mice (Figures 3F,G). Body weight (25.8 ± 0.7 vs. 24.8 ± 1.1 g), fasting blood glucose and fasting plasma insulin were unaffected (Figures 3H,I). This suggests that both overexpression and acute elevation of peripheral α-syn has a positive effect on glucose homeostasis. *In vitro* treatment of wild type isolated islets with α-syn monomers increased GSIS (Figure 3J), further confirming the ability of α-syn to directly stimulate insulin secretion from pancreatic beta cells.

### α-Syn Affects GLUT4 Trafficking

**FIGURE 4:**
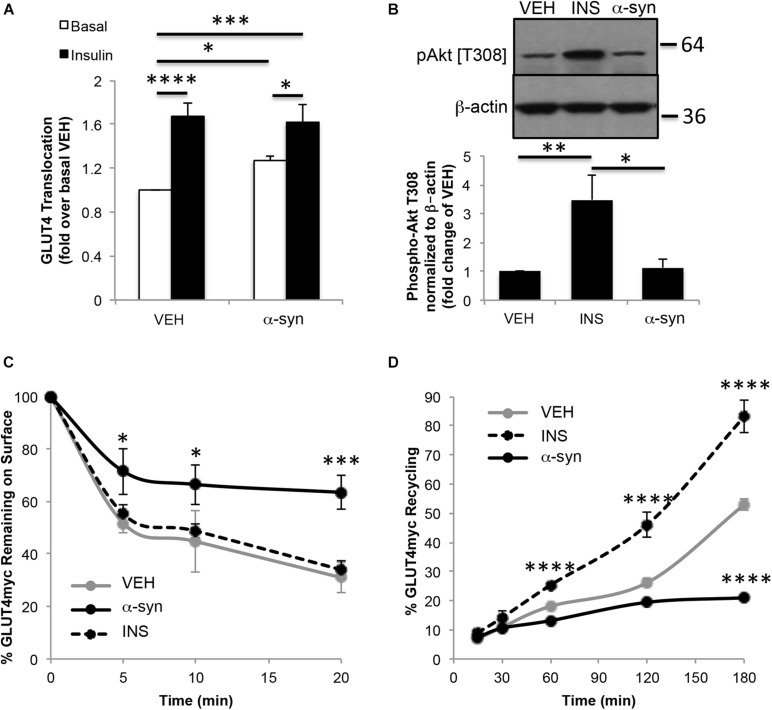
*In vitro* α-syn monomer treatment increases cell surface GLUT4 levels. **(A)** Surface GLUT4*myc* levels (*n* = 4–7), **(B)** Akt phosphorylation (*n* = 4), **(C)** GLUT4*myc* endocytosis (*n* = 4) and **(D)** GLUT4*myc* exocytosis (*n* = 3) in GLUT4*myc* L6 myotubes treated with vehicle, 100 nM insulin or 1.68 μg/mL α-syn monomers as described in Materials and Methods (^∗^*p* < 0.05, ^∗∗∗^*p* < 0.001, ^*⁣*⁣**^p < 0.0001).

Glucose uptake in skeletal muscle is mediated by GLUT4 glucose transporters. L6 myotubes stably expressing *myc*-tagged GLUT4 (GLUT4*myc*) are a cellular model used for studying GLUT4 traffic (Wang et al., 1998). Treatment of these cells with α-syn monomers resulted in a gain in GLUT4*myc* trafficked to the plasma membrane, which was not additive to the insulin response (Figure 4A). A previous report demonstrated that 3T3L1 preadipocytes exhibited a modest increase in Akt phosphorylation following treatment with α-syn, however, this was not observed in the current study (Rodriguez-Araujo et al., 2013; Figure 4B). The current findings indicate that α-syn in myotubes directly increases GLUT4 at the plasma membrane, independent of any change in Akt signaling. The impact of α-syn on GLUT4 trafficking was assessed further to determine if α-syn increases cell surface GLUT4 by enhancing GLUT4 exocytosis, reducing endocytosis or potentially via both of these pathways. We examined whether α-syn led to a reduction in GLUT4 endocytosis by measuring the surface GLUT4*myc* remaining at different times after internalization of labeled GLUT4*myc*. In both vehicle treated and insulin-stimulated states, GLUT4*myc* were internalized rapidly and to a similar extent under the two conditions (Figure 4C). In the presence of α-syn, GLUT4*myc* was largely retained at the cell surface, so that 64 ± 7% of the amount labeled remained associated with the surface after 20 min, compared with 31 ± 6% in the basal state. Conversely, to determine whether α-syn can boost the rate of GLUT4*myc* exocytosis, the time course of GLUT4*myc* appearance on the cell surface was detected in a live-cell recycling assay. The results showed a significant reduction at 180 min in cells stimulated with α-syn, while as previously reported, insulin increased GLUT4 exocytosis (Wijesekara et al., 2006; Figure 4D). Therefore, the α-syn-induced increase in surface GLUT4 levels is prominently associated with a reduction in GLUT4 endocytosis, while exocytosis is eventually reduced may be as a response to the impaired endocytosis. Cumulatively these data suggest that α-syn is also a key regulator of glucose uptake in skeletal muscle.

## Discussion

The aim of this investigation was to examine the physiological role of α-syn in glucose regulation. Our study demonstrated that α-syn is expressed in tissues important for glucose regulation such as pancreas and skeletal muscle, where it plays a role in maintaining insulin secretion and glucose transport.

Although α-syn expression was previously shown in pancreatic islets, its function in beta cells has been unclear (Sun et al., 2020). α-syn was suggested to be a cytoplasmic ligand of the insulin-secretory granule that interacts with the KATP channels to inhibit insulin secretion (Geng et al., 2011). In the current study, α-syn did not completely co-localize with insulin, suggesting that α-syn is not exclusively associated with the insulin granules. α-syn staining was frequently observed in the perinuclear region, indicating a possible association with the Golgi network where α-syn has been suggested to play a significant role in ER-to-Golgi transport and Golgi homeostasis (Wang and Hay, 2015).

Consistent with our results, a previous study found reduced density of surface insulin granules in α-synKO beta cells (Geng et al., 2011). Thus α-syn may also be involved in insulin granule trafficking within pancreatic beta cells. This is consistent with previous investigations, which have determined that α-syn is a microtubule binding protein (Alim et al., 2004; Cartelli et al., 2016). These findings support a contribution by α-syn to tubulin polymerization, which is an important requirement for insulin secretory activity. Alternatively, there is evidence of association of α-syn with secretary vesicles (Wang and Hay, 2015).

The function of α-syn in neurons has not been fully elucidated but, due to its presynaptic localization and interactions with membrane lipids, it has been hypothesized that α-syn plays a role in neurotransmitter release. Recently, α-syn by interacting with VAMP-2 has been reported to chaperone the assembly of the soluble N-ethylmaleimidesensitive factor attachment protein receptor (SNARE) complex, a process essential for membrane fusion events (Burré et al., 2010). In fact, triple-knockout mice for α-, β- and γ-synuclein exhibited decreased SNARE-complex assembly (Burré et al., 2010). SNARE complex involving syntaxin-1, SNAP25 and VAMP2 were reported to be important for mediating the release of insulin granules (Rorsman and Renström, 2003). It has also been suggested that α-syn plays a role in the stabilization of membrane curvature to prevent premature vesicle fusion and function as an anchor for vesicles at the plasma membrane (Lautenschläger et al., 2017). Cumulatively, these data suggest that there may be a defect in both insulin granule movement and exocytosis in α-syn knockout animals, leading to the reduction in GSIS and glucose intolerance observed in these mice.

It has been reported that elevated concentrations of serum α-syn are associated with increased insulin sensitivity in both humans and high fat fed rodents (Rodriguez-Araujo et al., 2015). We observed the development of peripheral insulin resistance in the absence of α-syn in knockout mice and improvement of insulin sensitivity when α-syn was overexpressed. A direct positive effect of α-syn on GLUT4 transloction was also observed in the absence of a change in insulin signaling, which is supported by a previous report showing an increase in glucose uptake in skeletal muscle upon intravenous administration of α-syn in wild type mice (Rodriguez-Araujo et al., 2013). The increase in GLUT4 at the plasma membrane in the presence of α-syn was attributed primarily to an inhibition of GLUT4 endocytosis. Recent studies have shown that α-syn plays a role in the endocytosis of synaptic vesicles with α-syn mutants or overexpression impairing endocytosis (Lautenschläger et al., 2017; Huang et al., 2019). Acute injection of lamprey synapses with α-syn inhibited both clathrin-mediated and bulk endocytosis from the plasma membrane during high-frequency stimulation (Busch et al., 2014). This study also suggested that excess α-syn may mask or mis-localize key lipids or proteins necessary for initiating synaptic vesicle recycling.

Interestingly, we observed a reduction in GLUT4 recycling at 180 min of α-syn stimulation compared to control. Since α-syn minimally affects GLUT4 exocytosis, we speculate that GLUT4 molecules responsible for α-syn-induced glucose transport originate from the same pool of transporters that maintain basal glucose uptake (i.e., the recycling endosomes). Thus the eventual reduction observed likely is in connection with reduced endocytosis, as this leads to the eventual depletion of the recycling pool.

In summary, the high prevalence of both T2D and PD warrants the need to understand further the causal relationship of the association for these two disorders. This study highlights a significant role for α-syn in glucose regulation, especially in beta cell function and glucose utilization in peripheral tissue, primarily relating to its role in vesicular trafficking. This signifies an important functional role of α-syn that is shared and common to the regulation of neurotransmission, insulin secretion and glucose uptake. Therefore, the current study emphasizes the need to gain more mechanical insight into the physiological function α-syn in the context of vesicular traffic in order to understand the causal link between T2D and PD.

## Data Availability Statement

The raw data supporting the conclusions of this article will be made available by the authors, without undue reservation.

## Ethics Statement

The animal study was reviewed and approved by University Health Network.

## Author Contributions

NW designed the experiments, acquired, analyzed and interpreted the data and drafted the manuscript. RA and TL contributed to the acquisition of the data. LW prepared α-syn monomers. PF and AT provided substantial contributions to the conception of the study and experimental design and interpretation of the data. All authors revised and approved the final version of the manuscript.

## Conflict of Interest

The authors declare that the research was conducted in the absence of any commercial or financial relationships that could be construed as a potential conflict of interest.

## Publisher’s Note

All claims expressed in this article are solely those of the authors and do not necessarily represent those of their affiliated organizations, or those of the publisher, the editors and the reviewers. Any product that may be evaluated in this article, or claim that may be made by its manufacturer, is not guaranteed or endorsed by the publisher.
